# Medical students as health coaches, and more: adding value to both education and patient care

**DOI:** 10.1186/s13584-017-0190-z

**Published:** 2017-11-30

**Authors:** Raymond H. Curry

**Affiliations:** University of Illinois College of Medicine, 1853 West Polk Street, MC 784, Chicago, IL 60612 USA

**Keywords:** Undergraduate medical education, Ambulatory education, Value-added medical education, Health coaches, Counseling for behavior change, Patient navigators

## Abstract

New ways of thinking about medicine and health care demand new methods in medical education. Over the past two decades, as both the practice and the study of medicine have become increasingly concerned with demonstrable outcomes, medical schools have developed new curricula in health systems science and are increasingly emphasizing students’ development and demonstration of skills essential to a systems-based, outcomes-oriented practice environment.

Polak and colleagues recently reported the development in Israel of one such curriculum, in lifestyle medicine, that includes opportunities for students to adopt the role of health coach. This commentary describes additional recent curricular developments elsewhere with similar goals, but utilizing more ambitious approaches that embed students in medical practices and provide meaningful, ongoing responsibility for assisting in the care of patients. These emerging new models for ambulatory care education, through a construct known as “value added education,” can simultaneously benefit both educational and patient care outcomes.

The ultimate goals of physician education are to be found in effective, safe and compassionate medical care. Whatever lens we look through – be it focused on ensuring the clinical skills of our students or on nurturing the pipeline for future investigators – the definitive end-product is patient care. Many recent developments in medical education recognize this fact, most notably the emphasis on Competency-Based Medical Education [1], an outcomes-based approach which is now informing curriculum development and assessment strategies at the medical school, residency, and continuing education levels.

In many ways, the development of this emphasis on educational outcomes has paralleled the focus on health care outcomes catalyzed by the Institute of Medicine’s 2001 *Crossing the Quality Chasm* report [2]. In its wake, we have seen the goals of medical care clarified and even redefined in several ways, bringing issues of patient safety, interprofessional collaboration, and systems-based practice to the forefront, along with the conceptual development of “health systems science” to stand alongside the traditional scientific and humanistic underpinnings of medical practice [3].

It should come as no surprise that these expectations for the outcomes of medical care and medical education are beginning to intersect in the classroom and in the clinic [4]. In a recent IJHPR article, Polak and colleagues [5] provide an example of a curriculum that helps students understand and begin to practice effective behavioral health interventions. Parenthetically, just as many recent developments in patient safety and patient-centered care have originated from a patient perspective, the curriculum described by Pollak et al. was initiated by one of the authors while a medical student.

The curriculum involves the incorporation of “lifestyle medicine” -- defined as the “evidence-based practice of assisting individuals and their families to adopt and sustain behaviors that can improve health and quality of life” [6] -- into multiple years of the MD curriculum at Hadassah-Hebrew University School of Medicine, using a hybrid model of lecture, case studies and group discussion, and experiential work by students. Each of the courses includes students’ participation as health coaches, addressing the needs of a patient (during the clinical years of the curriculum) or a family member or friend (during the preclinical years). The educational outcomes of the program described in this report are limited to analysis of the frequency and content of students’ coaching interactions and to student and faculty satisfaction with the curriculum, particularly the health coaching component. Initially introduced as an elective course sequence, its prompt acceptance by both students and faculty and its elevation to a mandatory component of the MD curriculum are clear indicators of success, as are early efforts to disseminate the model to other schools.

In reading this paper, however, I am struck by what seems a missed opportunity. Students in the preclinical years, and even some in the clinical years, did not have the opportunity to learn and hone their counseling skills by working with patients, but instead practiced with family members and friends. There are gains to be had by the “patients” as well as the students in this construct, to be sure – family and friends are simply patients, or patients-to-be, encountered from a different context. But some students did note a lack of fidelity to these encounters – inherent differences in the relationship with their “coachee” that made it more difficult for them to adopt the physician role, and affected their ability to practice the intended skills.

Moreover, educators are recognizing that there are additional advantages to be had when students are not only working with real patients, but are embedded in patient care activities and given responsibilities compatible with the knowledge and skills they have accrued, and can demonstrate, to date. The benefits are to be found in both educational and patient care outcomes [7]. For example, the “Education Centered Medical Home” at Northwestern University Feinberg School of Medicine in Chicago embeds teams of students, drawn from all years of the curriculum, in selected primary care settings for a biweekly clinic session. For the duration of their undergraduate medical education (four years, in the US curriculum), they work with the same group of peers, the same faculty preceptors, and the same panel of patients [8].

Given this level of continuity within a single care setting, students can do much more than simply practice clinical skills for their own benefit. They can be charged with a variety of responsibilities, such as contacting patients before and after an office visit and ensuring appropriate coordination of medications and referrals; they can also perform the role of health coach, as did the students in the work by Polak et al. [5] – and with the added opportunity to see the effects of these interventions over time. The work of Evans and Henschen et al. at Northwestern has demonstrated very promising educational outcomes, and further suggests that this model can directly improve the quality of patient care [8, 9].

Another similar innovation, and one more directly comparable to the course at Hadassah – Hebrew University School of Medicine, is the Systems Navigation Curriculum (SyNC) at Pennsylvania State University College of Medicine. Developed as a project within the American Medical Association’s Accelerating Change in Medical Education initiative [4, 10], the Penn State project embeds all first-year medical students in selected interprofessional, community-based clinical settings as patient navigators. Each student spends two to three half-days per month assisting patients with their understanding of the plan for their health care and their ability to carry it out – e.g., the logistics of their appointment schedules, tasks such as medication reconciliation, and basic health behavior counseling.

Both these efforts apply “value added” principles to medical education. The education of physicians has long included an experiential component – and Osler’s students were on the wards at Johns Hopkins several years before John Dewey’s advancement of modern experiential learning concepts. The accompanying potential for students to have a positive impact on care outcomes while they learn was noted some years ago [11], and recently expanded upon to make explicit an approach that has become known as “value added medical education” [12]. Along with the two examples cited here, others are now exploring the advantages of allowing even very early medical students to participate meaningfully, and productively, in patient care [7, 13–15].

In a broader sense, the value-added medical education concept addresses a fundamental issue that has bedeviled medical educators’ decades-long effort to provide more and better education in ambulatory settings. Osler’s now time-honored model for teaching on the inpatient wards involves students as an integral part of the patient care team, assuming progressive responsibility for various tasks and ensuring their presence during a variety of valuable learning opportunities. We have been slow to develop models of ambulatory and community-based education that are of similar value to the student. In fact, the most often cited barrier to effective ambulatory education is the impact upon the teaching faculty’s time efficiency and productivity – precisely because the student is not well enough integrated into the care setting to contribute to the work of the practice. Perhaps the secret to the value of ambulatory education for the student lies in the reciprocal value the student can bring to patient care.

This need for development of better models for ambulatory education is quite relevant to medical education in Israel. A 2014 external review of Israeli medical schools was critical of the slow adoption of ambulatory care education models, despite the country’s well-organized primary health care system. That review also noted the paucity of opportunities for students to actively practice clinical skills or engage with patients early in their medical education [16]. The further evolution of curricula along the lines described by Polak and colleagues [5] could contribute a great deal toward addressing these issues, adding value to both medical education and patient care in Israel.
